# Qualitative and Quantitative Detection of PrP^Sc^ Based on the Controlled Release Property of Magnetic Microspheres Using Surface Plasmon Resonance (SPR)

**DOI:** 10.3390/nano8020107

**Published:** 2018-02-13

**Authors:** Zhichao Lou, He Han, Dun Mao, Yibin Jiang, Jianyue Song

**Affiliations:** College of Materials Science and Engineering, Nanjing Forestry University, Nanjing 210039, China; 18805166615@163.com (H.H.); desmondgrant@163.com (D.M.); ybjiang97@163.com (Y.J.); sjy1005zy@163.com (J.S.)

**Keywords:** surface plasmon resonance 1, prion protein 2, aptamer 3, magnetic microsphere 4, self-assembling 5, sensitive 6

## Abstract

Prion protein (PrP^Sc^) has drawn widespread attention due to its pathological potential to prion diseases. In this work, we constructed a novel surface plasmon resonance (SPR) detection assay involving magnetic microspheres (MMs) and its controlled release property, for selective capture, embedding, concentration, and SPR detection of PrP^Sc^ with high sensitivity and specificity. Aptamer-modified magnetic particles (AMNPs) were used to specifically capture PrP^Sc^. Amphiphilic copolymer was used to embed the labeled PrP^Sc^ and form magnetic microspheres to isolate PrP^Sc^ from the external environment. Static magnetic and alternating magnetic fields were used to concentrate and control release the embedded PrP^Sc^, respectively. Finally, the released AMNPs-labeled PrP^Sc^ was detected by SPR which was equipped with a bare gold sensing film. A good linear relationship was obtained between SPR responses and the logarithm of PrP^Sc^ concentrations over a range of 0.01–1000 ng/mL. The detection sensitivity for PrP^Sc^ was improved by 10 fold compared with SPR direct detection format. The specificity of the present biosensor was also determined by PrP^C^ and other reagents as controls. This proposed approach could also be used to isolate and detect other highly pathogenic biomolecules with similar structural characteristics by altering the corresponding aptamer in the AMNPs conjugates.

## 1. Introduction

Prion protein (PrP^Sc^) has drawn widespread attention due to its pathological potential to prion diseases, such as Creutzfeldt Jakob syndrome and BSE [1]. PrP^Sc^ has strong resistance to heating, ultraviolet radiation, radiation and many chemical disinfectants, and general disinfection, freezing, drying and other similar procedures cannot make it lose toxicity [2]. In the meantime, during the long incubation period of the prion diseases (2 to 30 years), the virulent β-sheet rich PrP^Sc^ can introduce the transformation of the normal cellular PrP^C^ into PrP^Sc^ [3,4]. Therefore, there is an urgent need to develop a detection method of PrP^Sc^ for early diagnosis, which should be safe, sensitive and specific. 

Surface plasmon resonance (SPR) biosensor technology is a commercialized approach with a higher precision and sensitivity compared with traditional immunosorbent assays [5,6]. More and more researches have introduced SPR as a potential powerful tool for the detection of PrP^Sc^ and PrP^C^ [7,8,9]. However, it is reported that the minimum lethal dose of PrP^Sc^ in hamsters is less than 2 nM; the current sensitivity is not high enough for early diagnosis of prion disease [10]. In addition, due to the low molecular weight (23 kDa) and the trace amount of PrP^Sc^ mixed with the large volume of circulating blood, it is highly important for the creation of a novel SPR-based detection assay for the PrP^Sc^ detection with ultra-sensitivity and high-specificity. Recently, several approaches involving nanotechnology have been reported to enhance the SPR signals constructing a “sandwich” detection system [11,12]. 

There are still three challenges to overcome to realize the combination of SPR technology and nano-probe technology for the detection of PrP^Sc^: first, the designed biological nano-probe should have a highly specific property for the identification of PrP^Sc^ in the complex bio-samples, such as tissue, blood and organs; secondly, PrP^Sc^ should be isolated in the sample treatment procedures and released in the detection procedures, to avoid “second infection” and the possible effects of other substances (such as PrP^C^) coexisting in the samples on the detection results; finally, the structural characteristics of PrP^Sc^ should be fully utilized in the novel SPR systems to optimize the construction procedures of sensing film, reducing the detection costs and preventing the inevitable effects on the SPR detection results from the nonspecific interactions at the bio-nano interfaces [13]. 

**Figure 1:**
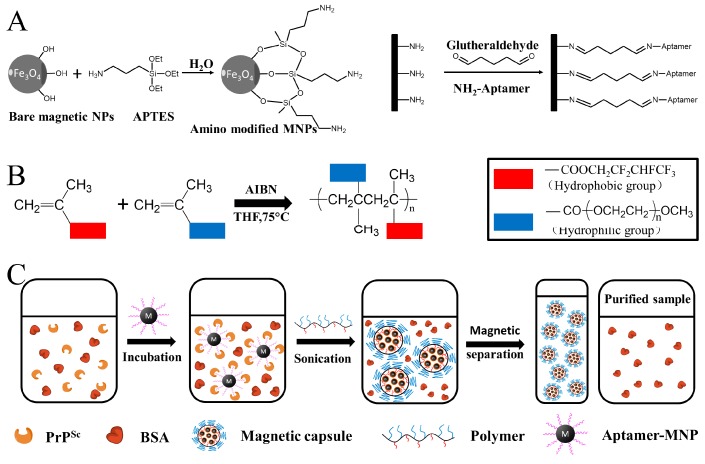
The schematic representation of (**A**) the synthesis processes of AMNPs, (**B**) the synthesis processes of amphiphilic copolymer, and (**C**) the capture, embedding and concentration processes of PrP^Sc^.

In this work, we constructed a novel SPR detection assay involving magnetic microsphere and its controlled release property, for selective capture, embedding, concentration, and SPR detection of PrP^Sc^ in the samples. Aptamer-modified Fe_3_O_4_ nanoparticles (AMNPs) synthesized through a silanization reaction and a glutaraldehyde (GA) crosslinking interaction (Figure 1A), are supposed to specifically capture the free PrP^Sc^ molecules in the sample (Figure 1C). Then, amphiphilic polymer (Poly(HFMA-g-PEGMA)) was synthesized through free radical reaction (Figure 1B) for the formation of magnetic microspheres (MMs) which were used to selectively embed the AMNPs labeled PrP^Sc^. The obtained MMs could be separated from the sample and enriched by a static magnetic field. By doing this, our purpose is to use MMs as a holder for the recognized free PrP^Sc^ molecules which are captured by AMNPs, to isolate them from the complex bio-environment, and to avoid “second infection” and the possible effects of other substances coexisting in the samples on the detection results. Our previous research confirmed that PrP^Sc^ molecules could self-assemble on gold surface via a coupling reaction between the disulfide bond and gold atoms [14,15]. Here the embedded PrP^Sc^ could be released under an alternating magnetic field just prior to the detection procedure and directly detected by SPR which is equipped with a bare gold film. This proposed approach could also be used to isolate and detect other highly pathogenic biomolecules with similar structural characteristics by altering the corresponding aptamer in the AMNPs conjugates.

## 2. Materials and Methods 

### 2.1. Materials

Prion protein was purchased from Calbiochem^®^ in Darmstadt, Germany (sequence: aa 23-231) and used as our previous work [14]. Terminal amino functioned SAF-93 [16] with 20 thymine bases as the spacer, which has been proven to have more than 10-fold higher affinity for PrP^Sc^ than for PrP^C^, were synthesized by Shanghai Sangon Biotechnology Co. in Shanghai, China. ThioPEG were purchased from Prochimia Surfaces in Gdansk, Poland. Cys-protein G (Catalog #: 1002-04) was obtained from Shanghai PrimeGene Bio-Tech Co. in Shanghai, China. Newborn Calf Serum (NBCS) was purchased from ThermoFisher Scientific (Waltham, MA, USA) (Catalog #:1610159). Other chemical reagents are supplied by Sigma with analytical purity. Human serum was purchased from Sigma-Aldrich (St. Louis, MO, USA).

### 2.2. Synthesis of AMNPs

APTES-Fe_3_O_4_ nanoparticles were firstly prepared according to the procedure described by previous reports (Figure 1A) [17,18]. Then the obtained APTES-Fe_3_O_4_ NPs (100 μL, 50 mg/mL) were redisposed in PBS (900 μL, 100 mM, pH 8.0) containing 0.2% (*v*/*v*) glutaraldehyde by ultrasonic dispersion for 20 min in room temperature followed by the addition of SAF-93 (100 μL, 40 μM). The glutaraldehyde crosslinking interaction was as in Figure 1A, under two-hour incubation with shaking (150 rpm) in room temperature. To remove the unmodified SAF-93 and excessive glutaraldehyde, the conjugates were collected by magnetic separation and washed by 100 mM PBS thrice. After that, the conjugates were further ultrafiltrated in a Nanosep 3K Omega filter device (filter size: 30 kDa).

### 2.3. PrP^Sc^ Labeling, Embedding, Enrichment and Release

The amphiphilic poly (HFMA-g-PEGMA) copolymers were firstly synthesized by free radical polymerization which is already published previously (Figure 1B, [19]). Then the capture and embedding processes of PrP^Sc^ are shown in Figure 1C. Briefly, 50 uL AMNPs solution (5 ng/mL) was firstly mixed with the samples (1 mL, in PBS buffer or NBCS) containing PrP^Sc^ in varied concentrations with other hybrid molecules (such as PrP^C^). Then the mixtures were incubated at 4 °C for 30 min. After the reaction, poly (HFMA-g-PEGMA) was added, and the mixtures were incubated in a water bath at 70 °C for 20 min under N_2_ protection. Finally, the synthesized MMs embedding the AMNPs labeled PrP^Sc^ were separated and concentrated in the presence of a static magnetic field. The purified samples were labeled as Sample B while the samples without any treatment were labeled as Sample A.

Then, the enriched MMs were re-dispersed in 1 mL PBS and incubated in an alternating magnetic field provided by an induction heating equipment (SPG-06-II, Shenzhen Shuangping Power Supply Technology Co. Ltd. (Shenzhen, China), frequency: 382 kHz, current intensity: 15 A). Because of magnetocaloric effect, the local temperature of MMs increased resulting in the damage of their structures and the release of PrP^Sc^. The concentrated AMNPs-PrP^Sc^ solution was labeled as Sample D.

### 2.4. Determination of the Induction Heating Time

During the incubation of MMs in the alternating magnetic field, a certain amount of the sample was taken every 5 min and treated by suction filtration using a permeable membrane with 30 nm pore size. The amount of iron in the filtrate was measured by the phenanthroline spectrophotometric method. Because the iron element present in the filtrate was derived from the AMNPs along with the released PrPSc, its amount reflected the release situation of PrP^Sc^.

### 2.5. In Situ SPR Measurement

The biosensor system used here is BI-2000 (Biosensing Inc., Tempe, AZ, USA). Prior to the detection, the bare gold film was immersed into the anhydrous acetone overnight to eliminate the possible contaminations. After repeatedly washing with water and drying with N_2_, the chip was annealed in a hydrogen flam. Then the sensing film was mounted on a SPR prism with the matching oil. Before the injection of the samples, the chip was rinsed with PBS and a stable baseline was obtained. Then the released AMNPs-labeled PrP^Sc^ in PBS was directly injected into the SPR cuvette for the detection. All the corresponding SPR signals were obtained in three repeated experiments independently, and the injection rate was set as 15 μL/min.

### 2.6. Characterization

The morphology of the nanoparticle was characterized by transmission electron microscopy (TEM) performed on a FEI Tecnai G^2^ 20 S-TWIN from FEI^®^ in Hillsboro, Oregon, USA. The IR spectra were recorded by FT-IR (FTIR920 from JingHe Analytical Instruments Company in Shanghai China), and each sample together with KBr was pressed to form a tablet. UV-Vis spectroscopy was carried out by a UV-3500 spectrophotometer (Shimadzu Corporation, Kotyo, Japan). Magnetic measurements were carried out using a Lake Shore 7407 VSM (East Changing Technologies, Inc., Beijing, China). 

AFM (Atomic Force Microscopy, Agilent Technologies, Santa Clara, CA, USA) was used to investigate the morphologies of the obtained AMNPs, MMs and the biosensing substrates before and after detection. An Agilent 5500 Controller combined with a 50 μm by 50 μm Agilent multipurpose AFM scanner was used to obtain high-resolution AFM images. Liquid phase AFM imaging was introduced for the observation of particles and the substrates, to avoid the negative effect of the possible physical agglomeration during the drying process on the morphology evaluation. In detail, 400 μL of each sample (particles) was deposited onto newly-caved mica surface in a liquid cell, and then the samples were imaged immediately at room temperature. For the substrates, they were imaged immediately after detection. Silicon cantilevers tip with spring constant of around 0.1 N/m were used. All the images were taken under TopMAC format with a PicoTREC controller (Agilent Technologies, Santa Clara, CA, USA). The 1024 × 1024 points AFM images were processed by WSxM software [20].

### 2.7. Safety Considerations

Prion protein is a potential infective pathogen. All the procedures of manipulating the PrP-containing samples should be done carefully and should comply with the American Centers for Disease Control (CDC) requirements for biosafety in microbiological and biomedical experiments. All the samples or solutions should be inactivated before removal from the laboratory by adding a sufficient volume of 8 M guanidinium chloride to the final concentration of 6 M. The mixture should be incubated at room temperature for at least 24 h to ensure that the infective molecules are inactivated. The solution of inactivated prions should be transferred to clean fresh containers and removed from the lab.

### 2.8. Detection of PrP^Sc^ in Real Sample

To demonstrate the potential application of this detection system, we detected PrP^Sc^ in human serum by using standard addition method. After mixing 5 ng/mL AMNPs with a series of standard solutions of PrP^Sc^ in non-diluted human serum by vigorous shaking. The mixtures were treated by the procedures as mentioned above, followed by in situ SPR measurements, and a calibration curve was constructed.

## 3. Results and Discussion

### 3.1. Characterization of AMNPs and Magnetic Microspheres

**Figure 2:**
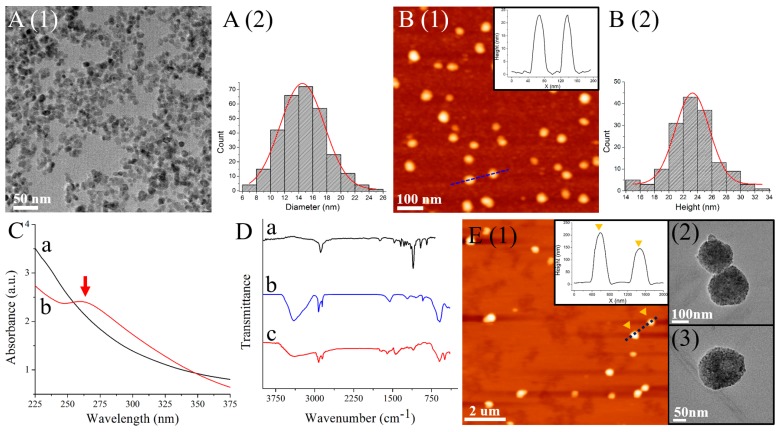
(**A**) TEM results of AMNPs and corresponding size distribution; (**B**) AFM results of AMNPs and corresponding size distribution; (**C**) UV-Vis spectra of APTES-Fe_3_O_4_ (black) and AMNPs (red); (**D**) FT-IR results of copolymer (black), AMNPs (blue) and MMs (red); (**E**) AFM results of MMs and the cross-section profile of the dashed blue line, and two zoom-in TEM images of MMs.

The morphology and structure of the synthesized AMNPs were examined by both TEM and AFM (Figure 2A,B, respectively). It is clearly observed that the size distribution obtained from TEM is 14.53 ± 0.08 nm, which is much smaller than the dimensions of AMNPs observed by AFM (23.29 ± 0.56 nm). This may be attributed to the presence of water molecules held by aptamers surrounding the Fe_3_O_4_ nanoparticles for the liquid AFM imaging. The conjugation of SAF-93 to Fe_3_O_4_ NPs could also be determined by the UV-Vis spectroscopy in Figure 2C. There is an obvious broad absorption band at around 260 nm in the spectrum of AMNPs, which is in accordance with the characteristic absorption band of RNA [21].

Figure 2D shows the FT-IR spectra of the poly (HFMA-g-PEGMA) copolymers (black), AMNPs (blue) and MMs (red). For AMNPs, the peak at 593.97 cm^−1^ is owing to Fe-O vibration. The peaks at 917.95, 1090.0 and 1051.5 cm^−1^ are owing to bending vibration of NH_2_ group, C-N stretching vibration, and Si-O stretching vibration, respectively. The presence of DNA-related peak at 1550.6 cm^−1^ and the peak at 1210.5 cm^−1^ attributes to the stretching vibrations of phosphate which is one of the three major components of RNA, indicating the successful modification of SAF-93 [22]. For the copolymers, the strong absorption peak at 1105 cm^−1^ is attributed to the CO characteristic stretching adsorption of PEGMA. The characteristic peak of C-F appears at 1342 cm^−1^ because of the HFMA segment. The peak at 1740 cm^−1^ can be attributed to the presence of C=O groups in both HFMA and PEGMA. The results indicate the synthesis of the amphiphilic copolymers. The appearance of the adsorption peaks at the similar wavenumbers compared to the FTIR results of both AMNPs and the copolymers, demonstrating the successful formation of MMs.

The amphiphilic poly(HFMA-g-PEGMA) copolymers show a tendency to form a core-shell microsphere in water. The water-soluble PEG chains (the blue segment in Figure 1B) serve as an outer hydrophilic shell stabilizing the microsphere. In addition, because of the hydrophobic characteristic of the HFMA segments and the intra-molecular β-sheets structure in AMNPs-labeled PrP^Sc^, they have a high tendency to bury themselves into the interior of the microspheres, forming a hydrophobic magnetic core. The morphology of the PrP^Sc^-encapsulated MMs is investigated by AFM and TEM in Figure 2E. From the AFM image, we may see that the MMs are stable with a narrow size distribution between 150 nm and 250 nm, and do not aggregate in water. In the TEM images, black clusters consisting of AMNPs and PrP^Sc^ are obviously observed.

### 3.2. Magnetic and Magnetocaloric Properties

**Figure 3:**
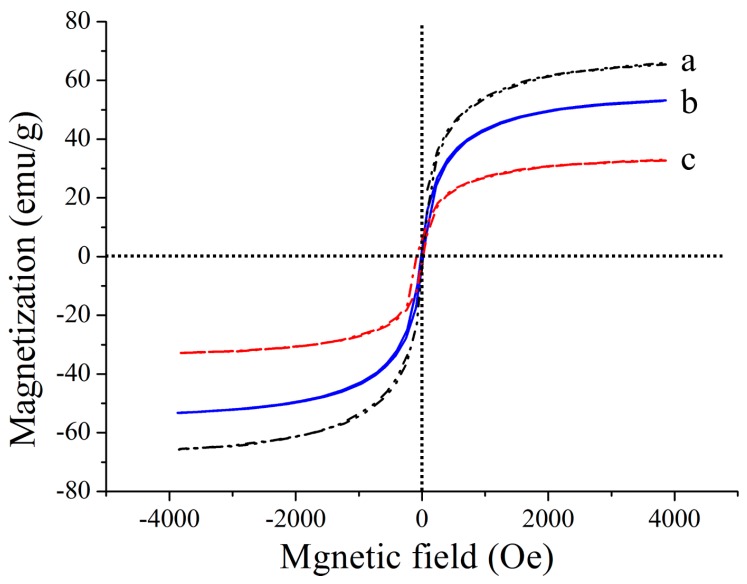
Magnetization hysteresis of Fe3O4 (black), AMNPs (blue) and MMs (red).

The magnetic properties of the synthesized Fe_3_O_4_, AMNPs and MMs were measured by vibrating sample magnetometer (VSM) (Figure 3). As could be seen, the typical characteristics of magnetic behavior are observed. The saturation magnetization values are 65.37, 53.20 and 32.24 emu/g. These results indicate that the magnetic microspheres dispersed in the solution have a rapid response in a static magnetic field, and the orientational enrichment of the imbedded PrP^Sc^ can be achieved in a short time.

**Figure 4:**
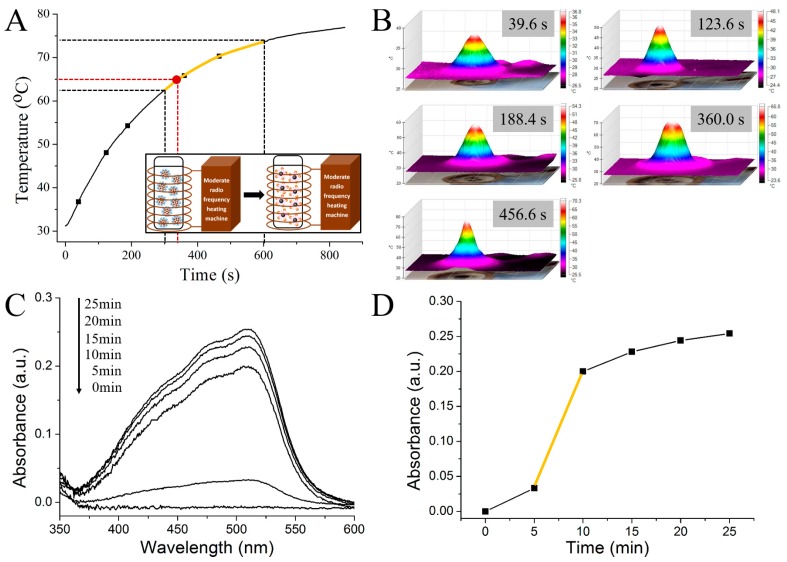
(**A**) Temperature vs. time curve of MMs; (**B**) Thermal images of MMs at time points in (**A**); (**C**) The orthophenanthroline spectrophotometry curves of iron elements in filtrates at time points; (**D**) The absorbance at 510 nm vs. time curve.

In a magnetic field produced by an alternating current coil (382 kHz, 15 A), the temperature of the magnetic microsphere varying with time is shown in Figure 4A. The temperature of the PrP^Sc^ embedded magnetic microsphere rises quickly at the beginning, and reaches 80 °C within 15 min. The three dimensional infrared thermal images in Figure 4B are consistent with the magnetothermal measurement results. Figure 4C is the ultraviolet absorption spectrum of the iron element, which is released from the magnetic microsphere in the samples at different incubation time in the alternating magnetic field. The corresponding obtained maximum absorption values of the spectrums at 526 nm varying with the incubation time are shown in Figure 4D. From the results, we may see that the amount of the released iron increases rapidly during the period between 5 and 10 min (the yellow part in Figure 4A,D), indicating that the damage of the structures of the magnetic microspheres caused by the magnetetocaloric effect begins at 5 min and nearly 80% of the embedded PrP^Sc^ molecules release from the microspheres in the following 5 min. Based on the results, 350 s was chosen as the induction heating time of the magnetic microspheres in the alternating magnetic field for the release of the embedded PrP^Sc^. The corresponding heating temperature is around 65 °C (identified in red in Figure 4A).

### 3.3. SPR Sensing for the Detection of Released AMNPs Labeled PrP^Sc^

**Figure 5:**
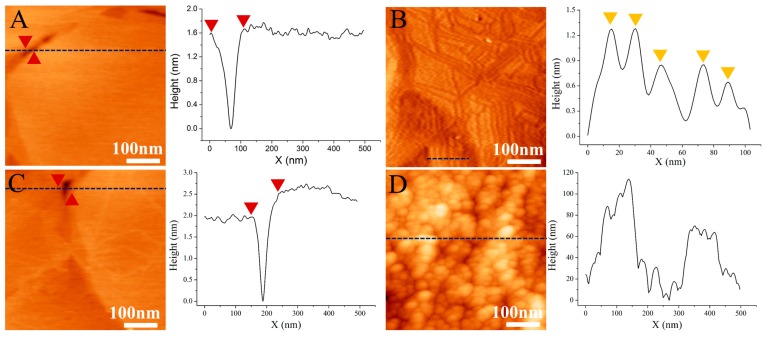
AFM images of (**A**) bare gold surface, and the surface morphology of the substrate (**B**) after direct detection of PrP^Sc^/BSA, (**C**) after the detection of the purified sample and (**D**) after the detection of the released AMNPs labeled PrP^Sc^.

As we discussed above, the PrP^Sc^ molecules were captured by AMNPs, embedded in the amphiphilic copolymer, and finally released with the help of alternating magnetic field. The released AMNPs labeled PrP^Sc^ were then detected in SPR equipped with bare gold sensing film. In order to test the isolation, enrichment and detection efficiencies of our designed processing and detection system for PrP^Sc^ in complex bio-samples, we began with examples of the PrP^Sc^/BSA mixed samples with the same concentration (50 ng/mL). As shown in Figure 5A, the bare gold surface is clean with characteristic gold edges. Figure 5B is the AFM results of the surface morphology of the SPR gold substrate after the detection of mixed PrP^Sc^/BSA sample. From the AFM image, we may see that the gold substrate is obviously modified by molecules yielding parallel linear patterns. From the corresponding cross-section files, we observe that all these molecules are of uniform sizes with the height values at around 1 nm, indicating that these observed molecules are formed by PrP dimers and trimmers as reported by our previous work [15], and BSA has no specific interaction with the sensing film. Figure 5C is the AFM image of the sensing substrate after the injection of the purified sample, which has isolated PrP^Sc^ via copolymer embedding and magnetic separation. The substrate is observed to be clean without any modification of PrP^Sc^, indicating that PrP^Sc^ are all isolated from the environment by the designed processes in this work. The AFM image of the surface morphology of the SPR sensing substrate after the injection of the released AMNPs-labeled PrP^Sc^, is totally different. As shown in Figure 5D, the obtained sensing film is uneven, with lots of dot-like particles modified on the surface. From the observed morphology and size under AFM, we may confirm that these particles are AMNPs which capture PrP^Sc^ and interact with the gold film via the Au-S bonding.

**Figure 6:**
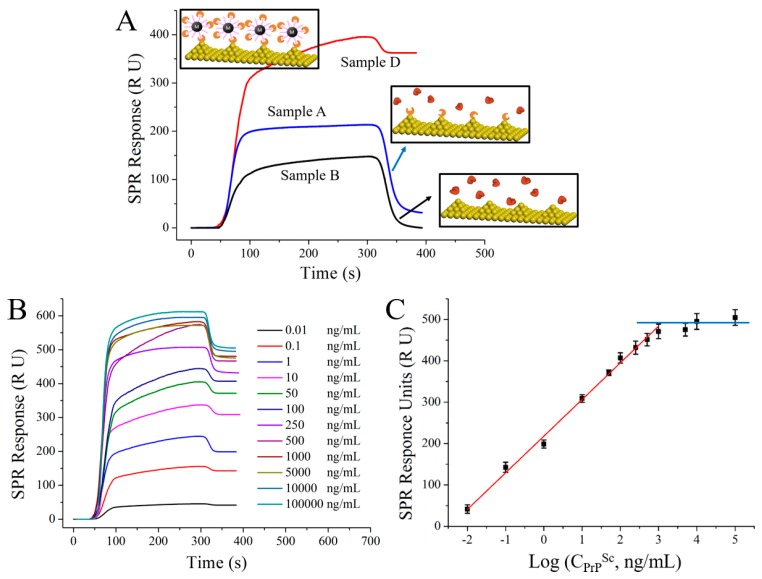
(**A**) The SPR detection signals of the treated (red), untreated (blue) and the purified (black) samples. Insert: Schematics of the SPR detections. (**B**) SPR sensorgram and (**C**) calibration curve of the SPR detection assay for PrP^Sc^ at different concentrations.

**Figure 7:**
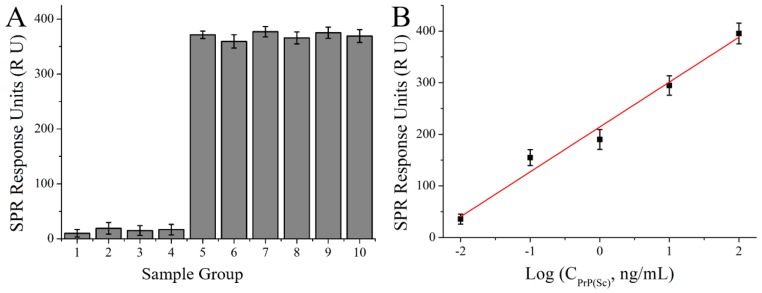
(**A**) Specific analysis of MMs involved SPR amplification detection. 1–4: MPA, thioPEG, Cys-protein G and PrP^C^ (50 ng/mL each) in PBS buffer, respectively; 5: PrP^Sc^ (50 ng/mL) in PBS buffer; 6: PrP^Sc^ (50 ng/mL) in NBCS; 7–10: Mixture of PrP^Sc^ (50 ng/mL) and each of the four different reagents (50 ng/mL) in PBS buffer. (**B**) Calibration curve of MMs involved SPR detection assay for PrP^Sc^ in human serum.

As we know that the SPR signal enhancement is certainly provided by the high refractive index and high molecular weight of the analyte, the state of the analyte captured on the surface of the sensing film and the resulting morphology should affect the detection signal [23]. As shown in Figure 6, the obtained SPR signals are 31.38 RU and 366.22 RU for the untreated and treated PrP^Sc^/BSA samples, respectively, while no SPR signal is obtained for the purified sample. All these results suggest that the designed analysis method here can isolate PrP^Sc^ from the complex biosystem with high specificity, and can increase the detection sensitivity (~10 fold). Figure 7A illustrates the variation of the obtained signals for the analysis of PrP^Sc^ in different concentrations. A good linear relationship is obtained between SPR responses and the logarithm of PrP^Sc^ concentrations over a range of 0.01–1000 ng/mL. The regression equation was *y* = 88.58*x* + 217.98 (*R*^2^ = 0.9943, *x* is the logarithm of PrP^Sc^ concentration (Log (ng/mL) and *y* is the SPR signal (RU)).

To fully evaluate the selectivity and specificity of the detection method here, PrP^Sc^ (50 ng/mL) in both PBS buffer and NBCS, three different reagents (MPA, thioPEG and Cys-protein G, 50 ng/mL) which all have sulfhydryl groups and can assemble on the gold surface, PrP^C^ (50 ng/mL), and the mixture of PrP^Sc^ (50 ng/mL) and each of the four different reagents (50 ng/mL) were measured respectively. The results are shown in Figure 7B, from which it can be observed that samples containing PrP^Sc^ have an average response of ~370 RU which is much greater than that of the other four samples, indicating the sufficient specificity of the treatment approach for PrP^Sc^ detection.

A calibration curve was obtained after detecting PrP^Sc^ in human serum using the MMs-involving SPR detection system constructed in this work (Figure 7B). The linear regression equation was *y* = 86.93*x* + 214.27 (*R*^2^ = 0.98, *x* is the logarithm of PrP^Sc^ concentration (Log (ng/mL)) and *y* is the SPR signal (RU)). RSD was 2.72% (*n* = 5) for 0.01 ng/mL PrP^Sc^. The corresponding recovery is 108.5%. This good linearity and recovery showed that the detection assay constructed here could be applied in detection of PrP^Sc^ in real sample.

## 4. Conclusions

This is the first report on the usage of MMs and their controlled release property, for selective capture, embedding, concentration, and SPR detection of PrP^Sc^ with high sensitivity and specificity. AMNPs were used for specific capture of PrP^Sc^ and for the amplification of detection signals. Amphiphilic copolymer was used to embed the labeled PrP^Sc^ and form magnetic microspheres to isolate PrP^Sc^ from the external environment. Static magnetic and alternating magnetic fields were used to concentrate and control release of the embedded PrP^Sc^, respectively. The released AMNPs-labeled PrP^Sc^ was detected by SPR which was equipped with a bare gold sensing film. A good linear relationship was obtained between SPR responses and the logarithm of PrP^Sc^ concentrations over a range of 0.01–1000 ng/mL. The detection sensitivity for PrP^Sc^ was improved by 10 fold compared with SPR direct detection format. The specificity of the present biosensor was also determined by PrP^C^ and other reagents as controls. This proposed approach could also be used to isolate and detect other highly pathogenic biomolecules with similar structural characteristics by altering the corresponding aptamer in the AMNPs conjugates.
